# The computation of confidence intervals for the state parameters of power systems

**DOI:** 10.1186/s40064-016-3631-1

**Published:** 2016-11-09

**Authors:** Zahid Khan, Radzuan Bin Razali, Hanita Daud, Nursyarizal Mohd Nor, Mahmud Fotuhi-Firuzabad, Katrina Lane Krebs

**Affiliations:** 1 Department of Fundamental and Applied Sciences, Universiti Teknologi PETRONAS, Tronoh Perak, 37150 Malaysia; 2 Department of Electrical Engineering, Universiti Teknologi PETRONAS, Tronoh Perak, Malaysia; 3 Department of Electrical Engineering, Sharif University of Technology, Tehran, Iran; 4 Higher Education Division, Central Queensland (CQ) University, Brisbane Campus, Brisbane, QLD Australia

## Abstract

**Background:**

In the past few decades, a significant volume of work has been carried out on various aspects of the state estimation problem to estimate an optimum state vector of the power system. This problem has been focused on, in previous studies regarding the computational efficiency and numerical robustness in view to find point estimates for system state parameters. This current investigation, constructed confidence intervals for the unknown state parameters of the system. The research indicates that confidence intervals can yield addition useful information about the estimated parameters.

**Methods:**

The feasible interval estimates for the system state parameters have been modelled in this study by considering the random uncertainty in the processing measurements. The statistical assumptions of the measurement errors have been utilized to characterize the probabilistic behavior of the estimated parameters in terms of confidence intervals. The Gauss–Newton algorithm has been adopted for maximizing the likelihood function of the processing measurements and obtaining the confidence intervals.

**Results:**

The usage of the confidence intervals was demonstrated through Monte Carlo experiments on a real dataset of the 6-bus and IEEE 14-bus power systems for both small and large sample sizes. The confidence intervals were constructed for the test networks for the sample of measurements 18, 28, 44 and 68 based on the redundancy ratio R. The proposed interval estimates outperformed for the sample sizes of 28 in the 6 bus and 68 in the IEEE 14-bus systems, respectively. The poor performance for the constructed interval estimates have been reported even for the large sample sizes in the existence of contaminated measurements.

**Conclusions:**

The results of the study show that the method is effective and practically applicable in the state estimation of a power system. The constructed confidence intervals for the system state parameters adequately perform for the lager sample size. However, the existence of the gross errors in the processing measurements had severe effect on the performance of the proposed interval estimates.

## Background

To achieve safe, effective and economic power delivery, it is mandatory for a control center to precisely estimate the existing state of the system. State estimation is, therefore, routinely practiced within the power industry for estimating the steady state of the system. The purpose of this estimation is to decrease the estimation error with an aim to increase the overall accuracy of the estimates. This estimation procedure is concerned with obtaining the point estimates of the state parameters. The probabilistic nature of the estimated parameters is however, not significantly addressed. The knowledge of the variability of these estimates can be served as additional information; so the reliable measure of the standard errors of these estimates is also of importance. This void forms the essence of the construction of confidence intervals for the state parameters reported in this work.

The output results of the state estimation algorithm are point estimates of unknown parameter values which are subsequently used in monitoring and planning the analysis of the system (Huang et al. 2012). Usually, these results are assumed to be reliable in the presence of random errors only for a fully observable system (MíDnguez et al. 2009). Providing the interval estimates along with single optimum estimates serves as additional information for an operator to enhance his/her confidence on the estimated results. The literature concerning the optimum single vector estimate for the state parameters is very rich and many algorithms have been proposed to this end, such as the weighted least squares (WLS) estimator (Caro et al. 2011) and robust estimators (Celik and Abur 1992; Mili et al. 1996; Baldick et al. 1997; Caro et al. 2013). Nevertheless, infrequent research has been carried out on state estimation with an aim to construct the interval estimates of state parameters. The work reported by Al-atwan and Koglin (1997) described the methodology for constructing the confidence intervals for the measurements used in the state estimation problem. The pioneering study of constructing confidence intervals for state parameters by considering the statistical uncertainty in measurements, is described in Kyriakides and Heydt (2006) for the linear functional forms of the measurement functions. Whereas, interval estimates for the nonlinear functional form are constructed by utilizing the concepts of the uncertainty analysis. The resulting intervals are then contained surely the true unknown state parameters (Kieffer and Walter 2005). Uncertainty concepts in estimation and optimization are basically by the work of Schweppe (1973) which have been extended also to other engineering areas (Brdys and Chen 1994). In state estimation context of the power system, the uncertainty study was implemented by Al-Othman and Irving (2005) by imposing double inequality constraints on each observation and then, solving the optimization problem. Later, more narrower deterministic interval estimates were presented by Wang et al. (2013) for conventional available measurements. In addition, the solution for the tightest lower and upper bounds have also been obtained for the state vector in the presence of synchronized measurements that are considered to be superior to conventional measurements (Rakpenthai et al. 2012). All these referenced interval estimates for state parameters, in the case of nonlinear measurement functions; rely strictly on the assumption of bounded errors. In this context, the uncertainty is bounded in input measurements to obtain deterministic confidence range of the upper and lower bounds on the estimated parameters. In reality, every measurement, especially in the case of a power system cannot be corrupted by the deterministic magnitude of the error then uncertainty in the parameters could be described stochastically by quantifying the probability distribution of the error terms. Moreover, one of the objectives of the state estimation is also the detection of bad data. Almost all the bad data techniques that are routinely implemented in state estimation rely on the usage of the Chi Square test statistic to decide the presence of bad data (Abur and Exposito 2004). The application of the Chi Square test is based on the assumption of the stochastic behavior of the noise terms.

Therefore, keeping in view the importance of the statistical assumptions of the error terms that are considered in the hypothesized nonlinear data generating model for conventional power measurements, this paper has described the confidence intervals for system state parameters. In the real world situation, all the observations required for estimating the state parameters are not available due to some reason, including the unavailability of telecommunication links or failure of devices etc. In the presence of the short availability of measurements, state estimation can be applicable however; these estimates are not as accurate to the actual values as the estimates based on fully observable systems. In order to enhance the operator’s confidence, on the results based on the state estimation; an inferential procedure can be implemented in the presence of the short availability of measurements. Confidence intervals are then the interval estimates which are most likely to include the true state vector which is composed of complex voltages at all nodes of the grid.

In the inferential view point, model assumptions of the noise terms play a key role for drawing the inference about unknown population parameters. In the statistical perspective, the state estimation of power system is a resemblance to the multiple nonlinear regression analysis with emphasis only on the point optimum estimate of the unknown state vector (Caro et al. 2013). Different approaches for obtaining the approximate standard errors could be used and are subsequently implied in the construction of approximate confidence intervals. The usages of each estimation method depend upon the problem at hand. In this case, linearization method for the construction of the approximate confidence intervals is applied because of its compatibility to the power flow study and it is less expensive in efficiency terms. Although none of the methods is deemed best practice and could lead to misleading results as pointed out by Tang and Yeh (2016). In the case of the nonlinear measurement functions, the linearization method is still superior in nonlinear estimation and has been extensively applied in different engineering problems (Cooley 1997; Vugrin et al. 2007).

The main contribution of this study is the application of the inference methodology in the state estimation environments because of the stochastic behavior of a power system. In reality, the state of the system is not stationary but varies instantly due to the change in load and other random disturbances. The statistical results of the estimation theory therefore, can be applied to describe the stochastic nature of the system state parameters.

## Methods

### Mathematical formulation of the problem

The observations are collected from the different measuring devices of the transmission system, inherently they are polluted because of the random noises and hence the actual value of any physical measure is not precisely known.

In mathematical form the problem can be written as

1 $$Z = h\left( X \right) + \varepsilon ,$$

where *Z* is *m*-dimensional vector of measurements, *h*(*X*) is a vector of nonlinear mean valued functions in terms of *k*-dimensional unknown state vector *X* and ε represents a Gaussian errors vector.

The details of this model is common in the technical literature on the power systems state estimation (Abur and Exposito 2004). However, the assumptions of the error terms used throughout this work are listed below:

*E*(ε) = 0 (mean vector), and Ω = *E*(εε^T^) (diagonal covariance matrix) with:

$$E\left( {\varepsilon_{i} \varepsilon_{j} } \right) = \left\{ {\begin{array}{ll} {\frac{{\sigma^{2} }}{{\sigma_{i}^{2} }}} \hfill &\quad {\forall i = j} \hfill \\ 0 \hfill &\quad { \forall i \ne j} \hfill \\ \end{array} } \right.$$

Here the term *σ*_*i*_^2^ is the weight, associated with the accuracy of *i*th measuring device and assumed to be known and σ^2^ represents the overall variance of the given measurements. For the construction of the individual confidence intervals, it is further assumed that the system is partially observable however; there is enough redundancy in measurements to estimate the system state parameters. Several least squares based methods for estimating the state parameters of a power system are conventionally used but the WLS approach performs the best in the estimation and identification of bad data (Habiballah and Irving 2000). In the form of the nonlinearity of *h*(*X*), the formulation of the confidence intervals could be straightforward but these intervals are not based on the standard Z, *T* and *F*statistical tests (Montgomery et al. 2012). Therefore, we rely on the results of the asymptotic theory that seem to be appropriate and have been used in such types of models in the context of other engineering problems (Cooley 1997). Due to the attractive large sample properties and great usefulness in drawing inferences in the case of nonlinear systems, we have considered the scoring method of maximum likelihood technique.

### Construction of the proposed confidence intervals

In this section, we have described the construction of the individual confidence intervals for the state parameters. The model given in Eq. (1) is the basic model that is used in the state estimation of the power system problem and structurally alike the nonlinear multiple regression model. In correspondence to the model given in Eq. (1), the transformed model is given by:

2 $$Z_{t} = h_{t} \left( X \right) + \varepsilon_{t},$$

where $Z_{t} = \varOmega^{{ - \frac{1}{2}}} Z,h_{t} \left( X \right) = \varOmega^{{ - \frac{1}{2}}} h\left( X \right),\varepsilon_{t} = \varOmega^{{ - \frac{1}{2}}} \varepsilon ,\varOmega^{{ - \frac{1}{2}}}$ is the Cholesky decomposition matrix of the weight matrix Ω^−1^ and $\varepsilon_{t} \sim N\left( {0, \sigma^{2} I} \right).$

Under the assumptions listed above, the log likelihood function for the observed sample is given by:

3 $$l_{n} \left( {X,\frac{{\sigma^{2} }}{Z}} \right) = constant + \frac{n}{2}logv - \frac{v}{2}\varphi \left( X \right)$$

where *φ*(*X*) = [*Z*_*it*_ − *h*_*it*_(*X*)]^*T*^[*Z*_*it*_ − *h*_*it*_(*X*)] and *v* = *σ* ^−2^.

In a power system state estimation, weights are known as nonnegative quantities which make us able to vary the influence of various measurements to the sum of squares. The maximum likelihood (ML) estimate of the scale parameter ν from Eq. (3) yields:

4 $$\hat{v} = \frac{n}{\varphi \left( X \right)}$$

Equation (3) is maximum when $\hat{\nu }$ takes the value as given in Eq. (4) for the fixed value of the state vector *X*. The concentrated likelihood function for the elements of *X* is therefore, given by:

5 $$l_{n} \left( X \right) = constant - \frac{n}{2}log \varphi \left( X \right).$$

The maximization of Eq. (5) is hence equivalent to the minimization of objective function φ(*X*) but, there is no closed form solution for obtaining the estimate of the parameter vector *X*. To tackle this nonlinearity in the iterative setting, we have been utilized the technique of profiling out the parameter ν from *l*_*c*_(*X*). In this way the method of the generalized least squares could be implemented more conveniently to evaluate the value of the optimum state vector *X* and the corresponding standard errors though a suitable iterative algorithm. The expected maximization algorithm, Fisher Information algorithm, and Newton–Raphson algorithm are the most common techniques for numerically maximizing the likelihood function (Wang 2007). In our case, Newton’s method and Fisher scoring produce the same results on account of the equivalence of the observed and expected Hessian matrix; so, we have been adopted Gauss–Newton algorithm for the solution of the nonlinear equations and finding the approximate standard errors.

Now the scoring function of Eq. (5) is given by:

$$\frac{{l_{c} \left( X \right)}}{\partial X} = - \frac{n}{2} \times \frac{1}{\varphi \left( X \right)} \times \frac{\partial \varphi \left( X \right)}{\partial X},$$

Setting the first order optimality conditions for maximizing Eq. (5) yields the set of *n* nonlinear equations in terms of unknown vector *X*.

6 $$- H_{t}^{T} \left( {Z_{t} - h_{t} \left( X \right)} \right) = 0 = S\left( X \right) ,$$

where $H_{t} = \frac{{\partial h_{t} \left( X \right)}}{\partial X}$ and *T* signifies transpose.

Let *X* ^*r*^ be the *rth* approximation state vector of the $\hat{X}$ then the Taylor series expansion of *S*(*X*) around *X* ^*r*^ is given by:

7 $$S\left( X \right) = S\left( {X^{r} } \right) + S^{\prime } \left( {X^{r} } \right)\left( {X - X^{r} } \right) + O\left( {X - X^{r} } \right) = 0,$$

where $$S^{\prime}\left( {X^{r} } \right) = \left. {\frac{\partial S\left( X \right)}{\partial X}} \right|_{{X^{r} }}$$ and $$r = 0, 1, 2, \ldots$$

Ignoring the higher order terms in Eq. (7), could yield the iterative solution for the point estimates. In order to find the standard errors of the estimates, we need to find the asymptotic variance covariance matrix for the estimated parameters.

In the given setting, we have the following observed Fisher Information matrix at the optimum state *X* ^*^:

8 $$\frac{{\partial^{2} l_{c} \left( X \right)}}{{\partial X\partial X^{T} }} = - \frac{{\hat{v}}}{2}\left[ {2H_{t}^{T} \left( {X^{*} } \right)H_{t} \left( {X^{*} } \right)} \right]$$

By using the ML estimate of *v* from Eq. (4) and the optimum value of *X* ^*^ from Eq. (7), we obtain the following observed Fisher Information matrix:

9 $$I\left( {X^{*} } \right) = - \frac{{\partial^{2} l_{c} \left( X \right)}}{{\partial X\partial X^{T} }} \approx \hat{v}\left[ {H_{t}^{T} \left( {X^{*} } \right)H_{t} \left( {X^{*} } \right)} \right] .$$

Hence, the asymptotic covariance matrix for estimated parameters becomes

10 $$\hat{V} = \hat{\sigma }^{2} \left[ {I\left( {X^{*} } \right)} \right]^{ - 1} ,$$

where *I*(*X* ^*^) = *H*_*t*_^*T*^(*X* ^*^)*H*_*t*_(*X* ^*^)and $\hat{\sigma }^{2} = \frac{{\varphi \left( {X^{*} } \right)}}{n - k}$ is the unbiased estimator of *σ* ^2^ and different from Eq. (4).

The value of $\hat{\sigma }^{2}$ in Eq. (10) is independently computed and it measures the degree of closeness of the measurements to the fitted equations at estimated value of *X*. Thus, the Gauss–Newton scheme for the individual estimates becomes

11 $$X^{i + 1} = X^{i} - \left[ {I\left( {X^{i} } \right)} \right]^{ - 1} S\left( {X^{i} } \right);\quad i = 1,2, \ldots$$

Equation (11) can be expressed equivalently in terms of the Gauss–Newton step as:

12 $$X^{i + 1} = X^{i} + \delta^{i} ; \quad i = 1, 2, \ldots$$

where *δ* = [*H*_*t*_^*T*^ *H*_*t*_]^−1^ *H*_*t*_^*T*^ *r*_*t*_ is called the Newton step.

The approximate Hessian matrix of the Gauss–Newton algorithm is *H*_*t*_^*T*^ *H*_*t*_ that may be infinite. This will eventually cause the Newton step in a non-descent direction. When this Hessian is in proximity to a singular form, the Gauss–Newton algorithm can yield a huge step which is often in a non-descent direction. The concept of this descent direction can be applied to establish the convergence of the Gauss–Newton algorithm. The convergence can be followed in fact by testing the following condition (Pajic and Clements 2005).

13 $$\left[ {H_{t}^{T} r_{t} } \right]^{T} \delta < 0 .$$

This condition has also been tested in the proposed method to assess whether the algorithm is leading to a descend direction or not. Consequently, a major reduction in the values of step norm δ and objective function *φ*(*X*) will be resulted by this descend direction. In general, the Gauss–Newton scheme is not essential to be in a descent direction due to the failure of the positive definite status of the Hessian matrix, consequently the iteration process may exhibit non-convergence (Chen 2011).

Thereafter, the linearized 100(1 − *α*)% confidence interval for the individual state parameter *x*_*i*_ can be specified as:

14 $$\hat{x}_{i} \pm t_{{\frac{\alpha }{2}, \left( {n - k} \right)}} \sqrt {\theta_{ii} } ,$$

where $\theta_{ii}$ is the *i*th diagonal element of the matrix given Eq. (10) in correspond to the estimate *x*_*i*_, $t_{{\frac{\alpha }{2}, \left( {n - k} \right)}}$ is the percentage point for the student *t* − distribution and (*n* − *k*) is the degree of freedom. The primary assumption of using the linearized confidence intervals (LCIs) is that the measurements are randomly and independently sampled from the measuring devices. The validity of these intervals is supported by the results of the asymptotic theory. The intervals calculated in this way are approximated. Their adequacy depends on the good linear approximation to nonlinear functions which means that the effect of the curvature and the higher terms of the Taylor expansion would be insignificant and to that of the asymptotic result ($\hat{X} \sim N_{k} \left( {X,\sigma^{2} \left[ {I\left( X \right)} \right]^{ - 1} } \right)$. This asymptotic result holds under the appropriate regularity conditions (Seber and Wild 1989). In power flow analysis, structures of nonlinear measurement functions *h*(*X*) for the real and reactive powers are common and well established (Khaitan et al. 2010). The influence of the curvature and higher order terms in linearizing the nonlinear functions involved in the iterative scheme are negligible in practice (Van Amerongen 1995). Thereby, the first order Taylor approximation in the proposed LCIs is sufficed for linearizing these nonlinear measurement functions. Additionally, as we are assuming that the actual value of the state vector *X* is not known, therefore, the Fisher matrix is estimated by $I\left( {\hat{X}} \right)$ and *σ* ^2^ as given in Eq. (10). The estimation procedure for the state parameters can be embodied in the Gauss–Newton algorithm as follows. 

The aforementioned algorithm is continuously applied in the iterative setting until there is no essential change between the successive values of X being observed from one iteration to the next iteration.

### Simulation experiment and results

In this section, we have developed the simulation experiment for our described intervals in order to check their validity in the view to implement them in the state estimation environments of the power system. For implementation purposes, we have been taken the data of the 6-bus and IEEE 14-bus power systems. The main characteristics of the test networks are shown in Table 1, whereas the full set of measurements and system’s configuration can be found (Christie 2000; Wood and Wollenberg 2012).

**Table 1:** Main characteristics of the 6-bus and IEEE 14-bus power networks

Characteristics	6-Bus power system	IEEE 14-bus power system
Number of nodes	6	14
Number of lines	11	20
Full set of measurements	62	122
Parameters to be estimated	11	27

It is assumed that all the measurements for estimating the state vector *X* are not available but there is enough redundancy in the measurements to the find interval estimates of the actual values. The adequacy of these intervals has been evaluated on the basis of the coverage probability. To find the impact of the sample size on the calculated coverage probabilities, we have considered the different sample sizes with respect to the unknown parameters in *X*. There is no hard and fast rule for the selection of a suitable sample size for the nonlinear least squares problems. However, we have categorized the size as small and large in accordance with the redundancy ratio that is defined as below for the system having *N* nodes and *B* branches:

15 $$R = \frac{3N + 4B}{2N - 1}$$

Here, 3 *N* + 4*B* is the total number of observations for the fully observable system and 2 *N* − 1 represents the number of parameters in *X*. The vector of the unknown parameters is consisted of all voltage magnitudes for the *N* nodes and the *N* − 1, phase angles except for the angle of the slack bus which is always assumed to be zero to address the problem of the power losses (Dimitrovski and Tomsovic 2005). In mathematical perspective, this assumption is allowed for the feasible solution of the nonlinear set of equations given in (1). The solvability of the algorithm for point estimates and their respective standard errors requires at least 2 *N* − 1 measurements. The program for the proposed methodology has been written in MATLAB environment. We set the simulation study for the 6-bus system but same discussion is held for the IEEE 14-bus system. In our simulation experiment, we observed the reasonable width of our calculated intervals for *R* ≥ 2.5. Therefore, we have presented our results for the samples of sizes 18 and 28 for the 6-bus network with redundancy ratio of less than and greater than 2.5 respectively. The sample of the 18 observations was consisted of all the voltages, the real and reactive power injections whereas, the sample of 28 observations contained all the voltages, and the real and reactive power injections and five measurements each on the real and reactive power flows of the system. In order to evaluate the performance of the confidence intervals as given in Eq. (11), the true or base value of the state vector *X* was required. For this purpose, the system was solved by using the MATPOWER 5.1 and the output for the state vector *X* based on 62 measurements was assumed to be the actual values of the system. For the usage of WLS approach, it assumed that the source of this uncertainty was mainly added in the actual measurements by the expected accuracy standards of the measuring devices which were installed at different locations. The variability in the measurements that was caused by these measuring instruments is according to the following variability standards as shown in Table 2.

**Table 2:** Variability in the measurements from different measuring devices

Measurements	Standard deviations in actual units
*P*_*inj*_, *P*_*flow*_	3 *MW*
*Q*_*inj*_, *Q*_*flow*_	3*MVAR*
*V*_*mg*_	2 *kV*

This data set was then perturbed by adding the error terms according to model Eq. (1). Each noise term had been taken from the Gaussian distribution with the mean zero and standard deviation in correspondence to the assumed accuracy standard used for the respective measuring device as shown in Table 2. In this way, a set of 18 noisy measurements was used in calculating the point estimates through the Gauss–Newton algorithm and the respective standard errors were obtained at the specified tolerance *ɛ*, which was chosen as 10^−4^.

Since the convergence of the algorithm is highly associated with the reasonable start of the unknown vector, we therefore, initialized the state vector with a routinely flat set, i.e., zero for each angle and one for every voltage in *X*. This is routinely exercised in a power flow analysis to express the measurements in per unit system (Abur and Exposito 2004). The per unit means that 100 MV, 100 MVAR and 230 kV are used as common bases to covert the actual measurements of the real power, reactive power and voltages into a per unit system. This is more a convenient way for simulation and general practice in the power flow study. The simulation was run 10,000 times to find intervals for each parameter according to Eq. (14). The results of the coverage probabilities for both sample sizes are reported in Tables 3 and 4 at the nominal coverage probability of 95%.

**Table 3:** Performance of the interval estimates for the 6-bus system parameters (*n* = 18)

Estimated parameters	True values	Average LCL	Average UCL	Coverage Prob (%)
*θ*_02_	−3.70	−3.98	−3.36	94.20
*θ*_03_	−4.30	−4.78	−3.76	94.11
*θ*_04_	−4.20	−4.49	−3.89	94.12
*θ*_05_	−5.30	−5.66	−4.89	94.26
*θ*_06_	−5.90	−6.46	−5.43	94.62
*V*_01_	241.50	237.41	243.66	94.12
*V*_02_	241.50	239.63	243.42	94.12
*V*_03_	246.10	244.33	249.49	94.12
*V*_04_	227.60	225.38	229.78	94.02
*V*_05_	226.70	224.78	229.65	94.72
*V*_06_	231.00	228.86	233.20	94.14

*LCL* lower confidence limit, *UCL* upper confidence limit

**Table 4:** Performance of the interval estimates for the 6-bus system parameters (*n* = 28)

Estimated parameters	True values	Average LCL	Average UCL	Coverage Prob (%)
*θ*_02_	−3.70	−3.91	−3.42	94.76
*θ*_03_	−4.30	−4.68	−3.85	94.94
*θ*_04_	−4.20	−4.43	−3.95	94.76
*θ*_05_	−5.30	−5.58	−4.96	95.00
*θ*_06_	−5.90	−6.36	−5.52	95.06
*V*_01_	241.50	239.67	243.39	95.08
*V*_02_	241.50	239.82	243.22	94.70
*V*_03_	246.10	244.07	248.14	95.02
*V*_04_	227.60	225.65	228.56	94.76
*V*_05_	226.70	224.56	228.80	95.12
*V*_06_	231.00	229.13	232.98	94.96

Likewise, the individual confidence intervals have also been reported in the Tables 5 and 6 for the IEEE 14-bus system parameters for the samples of sizes 44 and 68 respectively in accordance to the same system’s redundancy ratios.

**Table 5:** Performance of the interval estimates for the IEEE 14-bus system parameters (n = 44)

Estimated parameters	Base case values	Average LCL	Average UCL	Coverage Prob (%)
*θ*_02_	−04.98	−05.14	−04.75	97.22
*θ*_03_	−12.72	−13.18	−12.11	97.94
*θ*_04_	−10.33	−10.64	−09.75	97.92
*θ*_05_	−08.78	−09.11	−08.30	98.24
*θ*_06_	−14.22	−15.36	−13.09	97.84
*θ*_07_	−13.37	−14.11	−12.14	98.30
*θ*_08_	−13.36	−14.48	−11.72	97.94
*θ*_09_	−14.94	−15.74	−13.62	98.04
*θ*_10_	−15.10	−16.08	−13.75	97.80
*θ*_11_	−14.79	−15.98	−13.47	97.98
*θ*_12_	−15.07	−16.66	−13.62	96.96
*θ*_13_	−15.16	−16.53	−13.83	98.14
*θ*_14_	−16.04	−17.38	−14.56	97.02
*V*_01_	243.80	242.05	246.75	95.78
*V*_02_	240.35	238.72	243.14	95.56
*V*_03_	232.30	230.28	235.48	96.56
*V*_04_	234.14	232.55	236.29	97.48
*V*_05_	234.60	233.07	236.87	97.04
*V*_06_	246.10	244.57	248.74	95.92
*V*_07_	244.26	241.50	245.61	93.60
*V*_08_	250.70	247.68	254.17	97.76
*V*_09_	242.88	238.65	243.67	80.58
*V*_10_	241.73	238.34	242.95	89.72
*V*_11_	243.11	240.71	245.76	97.98
*V*_12_	242.65	240.19	246.38	97.12
*V*_13_	241.50	239.66	244.63	95.98
*V*_14_	238.28	235.02	241.00	97.76

**Table 6:** Performance of the interval estimates for the IEEE 14-bus system parameters (n = 68)

Estimated parameters	Base case values	Average LCL	Average UCL	Coverage Prob (%)
*θ*_02_	−04.98	−05.45	−04.51	94.96
*θ*_03_	−12.72	−13.26	−12.19	94.82
*θ*_04_	−10.33	−10.85	−09.78	95.22
*θ*_05_	−08.78	−09.26	−08.30	94.70
*θ*_06_	−14.22	−14.82	−13.61	94.66
*θ*_07_	−13.37	−13.96	−12.75	95.08
*θ*_08_	−13.36	−14.05	−12.65	94.44
*θ*_09_	−14.94	−15.55	−14.31	94.94
*θ*_10_	−15.10	−15.88	−14.30	94.60
*θ*_11_	−14.79	−15.62	−13.94	95.30
*θ*_12_	−15.07	−16.86	−14.03	94.74
*θ*_13_	−15.16	−16.05	−14.24	94.74
*θ*_14_	−16.04	−16.97	−15.09	95.20
*V*_01_	243.80	239.82	247.85	94.54
*V*_02_	240.35	236.44	244.19	94.72
*V*_03_	232.30	228.48	236.12	95.10
*V*_04_	234.14	230.29	237.98	94.72
*V*_05_	234.60	231.01	238.32	95.12
*V*_06_	246.10	242.53	249.66	94.70
*V*_07_	244.26	240.20	248.24	94.98
*V*_08_	250.70	246.69	254.74	95.10
*V*_09_	242.88	239.13	246.65	95.22
*V*_10_	241.73	237.71	245.76	94.82
*V*_11_	243.11	239.07	247.13	95.06
*V*_12_	242.65	238.96	246.27	94.68
*V*_13_	241.50	237.84	245.23	94.66
*V*_14_	238.28	234.20	242.26	94.84

The sample of 44 observations was comprised of all voltage magnitudes; all measurements on real and reactive power injections and one measurement each on real and reactive power flows. Whereas, the sample of 68 contained all voltage measurements, all measurements on the real power injections and the real power flows.

## Discussion of the results

The coverage probability is the proportion of the samples which are taken with repeated sampling to the total number of samples that contain the true value of the population parameter. It is used as one of criteria for the evaluation of the confidence intervals. Intervals are said to be exact if (1 - *α*)100% of such intervals include the true parameter; otherwise, the intervals would be characterized as liberal or conservative if the coverage probability remains below or exceeds the nominal probability (1 - *α*) respectively. We have explored the performance of the LCIs for the state vector parameters for partially observable systems. It has been seen in Table 3, the results of the approximate coverage probabilities for the desired parameters in the presence of a small sample is good and closed to the nominal coverage of 95%. We constructed the confidence intervals for a larger sample, as shown in Table 4, the coverage probabilities move closer to the nominal value for the liberal and conservative confidence intervals. So, the overall performance of the approximate confidence intervals is better irrespective of the large or small sample sizes. However, this is not true in general because the performance of the LCIs are strictly based on the validity of the linear approximation algorithm. In addition to the nonlinearity of the model, and the approximation of the Hessian matrix, larger residuals could result in a suspicious confidence region. For the 6-bus system, the impact of the residual mean square is less and we have been obtained analytically derivatives for the approximation of the Hessian matrix in our algorithm. This resulted in better coverage probabilities even in the case of the short redundancy. In order to explore more, the Gauss–Newton algorithm has been run on the IEEE 14-bus power system to find the adequacy of the LCIs for the state vector parameters. The simulation output of the coverage probabilities with the small and large samples are shown in Tables 5 and 6, respectively. We observed the poor performance of the LCIs in the case of the small sample size and most of the intervals for the desired parameters are liberal; whereas, the approximate confidence intervals performed adequately in the case of the large sample size. The fact behind the discriminate performance in this case, was the large impact of the residuals on the standard errors of the estimated parameter for the smaller sample. Therefore, the overall performance of the approximate confidence intervals was good for the larger samples in terms of coverage probabilities.

**Fig. 1:**
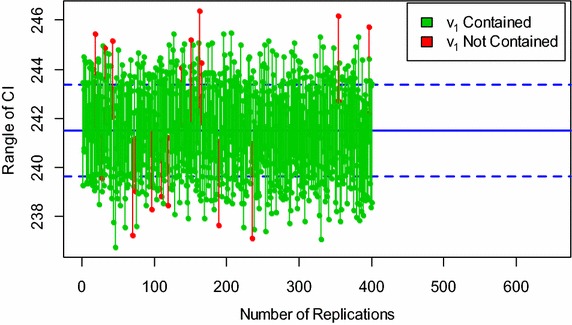
Simulation of the LCIs for the parameter ν_1_ of the 6-bus system at n = 28

**Fig. 2:**
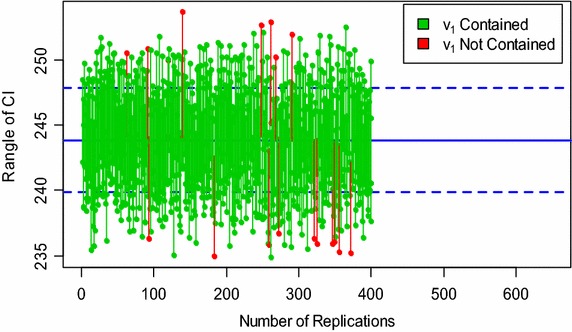
Simulation of the LCIs for the parameter ν_1_ of the IEEE 14-bus system at n = 68

The procedure of calculating the desired intervals up to 400 runs can also be seen in Figs. 1 and 2 for the parameter *v*_1_ (i.e., voltage at node one) of the 6-bus and IEEE 14-bus systems, respectively.

The central parallel lines in each graph correspond to the actual values of the estimated parameter *v*_1_ of the 6-bus and IEEE 14-bus networks. Each vertical line that is across the central parallel lines is equivalent to the interval that holds the true value of the parameter. Whereas, the other vertical lines that are below and above the central lines are equivalent to the intervals that do not contain the true parameter $v_{1}$.

**Fig. 3:**
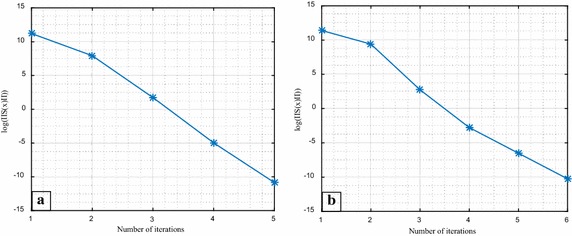
The convergence of the Gauss–Newton algorithm for; **a** the 6-bus system parameters, **b** IEEE 14-bus system parameters

Note that, the condition given in Eq. (13) had been applied successfully for different sample sizes of the 6-bus and IEEE 14-bus networks’ data in order to guarantee the convergence of the Gauss–Newton algorithm. However, to save the space, the iteration results for the sample size of 28 measurements in the case of the 6-bus system and the sample size of 68 for the IEEE-14 bus have been considered to view the convergence feature of the Gauss–Newton algorithm. A seed was set in the algorithm in order to retain a fixed random vector for the perturbed measurements. The convergence performance of the algorithm for both test cases are shown in Fig. 3.

Figure 3 illustrates that the iterative procedure in either case was proceeding to the right direction. In addition, the behaviors of the norm steps during the iteration process in each test case and corresponding reduction in their loss functions are given in Table 7.

**Table 7:** The Gauss–Newton algorithm applied to the 6-bus and IEEE 14-bus test systems

6-bus system			IEEE 14-bus system		
Iterations	*φ*(*X*)	*δ*	Iterations	*φ*(*X*)	*δ*
1	7.8904 × 10^3^	0.2223	1	1.850 × 10^4^	0.9305
2	21.6772	0.0120	2	178.5464	0.0840
3	7.2942	3.6055 × 10^−4^	3	25.0504	2.2321 × 10^−4^
4	7.2941	2.8976 × 10^−5^	4	25.0496	5.5801 × 10^−6^
5	7.2941	2.3286 × 10^−10^	5	25.0496	1.1191 × 10^−7^
–	–	–	6	25.0496	2.9793 × 10^−9^

The results in Table 7 are also in accordance with the gradient behaviors shown in Fig. 3 which confirms that the descend directions ultimately caused the reduction in both norm step and the loss function.

Here, we are assuming that our available data vector did not contain any gross errors as these calculated intervals might not be robust in the presence of bad data. The existence of outlier values may be rigorously impaired the estimation results and needs an adequate caution to be taken. This fact has been studied by many researchers in view of point estimation for the unknown state parameters (Anwar and Mahmood 2014). To this reason end, the performance of the proposed interval estimation had also been carried out in the presence of contaminated measurements.

Incidentally, the data generating model for the contaminated measurements in accordance with Eq. (1) can be written as follows:

16 $$Z = h\left( X \right) + \delta \vartheta + \varepsilon ,$$

where *δ* is magnitude of gross error in some multiple of standard deviation i.e., *δ* = *kσ*_*i*_ and *ϑ* is a vector of zero elements except one at the *ith* position for the gross error in the measurement from the *ith* measuring device. To determine the robustness of the calculated intervals, a bad measurement was interrupted at the real power injection at node one by adding the four times standard deviation of its measuring instrument according to Eq. (16). The results are shown in Tables 8 and 9 for the 6-bus and IEEE 14-bus systems respectively.

**Table 8:** Performance of the interval estimates for the 6-bus system parameters in the presence of a single bad value (*n* = 28)

Estimated parameters	True values	Average LCL	Average UCL	Coverage Prob (%)
*θ*_02_	−3.70	−4.82	−3.26	97.12
*θ*_03_	−4.30	−6.10	−4.12	81.56
*θ*_04_	−4.20	−5.29	−4.10	89.56
*θ*_05_	−5.30	−6.67	−5.23	80.71
*θ*_06_	−5.90	−7.68	−5.11	60.67
*V*_01_	241.50	238.86	245.72	98.12
*V*_02_	241.50	238.22	244.54	98.54
*V*_03_	246.10	242.57	249.26	99.10
*V*_04_	227.60	224.04	231.18	98.07
*V*_05_	226.70	223.06	230.11	96.23
*V*_06_	231.00	227.23	234.36	97.44

**Table 9:** Performance of the interval estimates for the IEEE 14-bus system parameters in the presence of a single bad value (*n* = 68)

Estimated parameters	True values	Average LCL	Average UCL	Coverage Prob (%)
*θ*_02_	−04.98	−06.00	−04.53	93.48
*θ*_03_	−12.72	−14.07	−12.40	84.24
*θ*_04_	−10.33	−11.70	−10.02	82.08
*θ*_05_	−08.78	−10.13	−08.62	72.62
*θ*_06_	−14.22	−15.86	−13.98	71.46
*θ*_07_	−13.37	−14.93	−13.03	72.92
*θ*_08_	−13.36	−15.08	−12.90	89.54
*θ*_09_	−14.94	−16.55	−14.60	84.48
*θ*_10_	−15.10	−16.98	−14.53	91.58
*θ*_11_	−14.79	−16.77	−14.16	92.24
*θ*_12_	−15.07	−17.36	−14.17	95.14
*θ*_13_	−15.16	−17.24	−14.44	93.76
*θ*_14_	−16.04	−18.17	−15.26	94.02
*V*_01_	243.80	237.11	249.54	99.16
*V*_02_	240.35	233.53	245.50	98.64
*V*_03_	232.30	226.27	238.08	97.68
*V*_04_	234.14	228.30	240.20	98.62
*V*_05_	234.60	230.01	241.28	98.12
*V*_06_	246.10	240.84	251.87	97.60
*V*_07_	244.26	237.96	250.38	98.64
*V*_08_	250.70	244.48	256.95	98.82
*V*_09_	242.88	236.90	248.53	98.70
*V*_10_	241.73	235.50	247.94	98.74
*V*_11_	243.11	236.85	249.31	98.74
*V*_12_	242.65	237.98	248.30	98.74
*V*_13_	241.50	235.80	247.23	98.75
*V*_14_	238.28	232.00	244.46	98.78

It can be observed that even for the large samples, the performance of the approximate intervals is very poor in the presence of a gross error. This is because of the fact that the inclusion of a single bad value, although only causing a slight, increased in the standard errors from the Fisher matrix, but substantially enlarged the mean standard errors which in return causes the width enlargement for the approximated intervals.

## Validity and comparison of the proposed confidence intervals

At the end of this analysis, the question also arises about the correctness of proposed interval estimates. Thereby, in view to verify further the correctness of proposed method for the construction of individual confidence intervals, we have taken the results of the tightest upper and lower bounds obtained by Al-Othman and Irving (2005) for the unknown state parameters of the 6-bus system. The numerical simulation results in the aforementioned work of Al-Othman and Irving (2005) were obtained by assuming bounded errors in each measurement within the range of [−3, 3%]. The nonlinear optimization problem with double inequality was then solved in order to calculate the uncertainty intervals of the system state parameters. We took the uncertainty intervals as known interval estimates for the true unknown parameters in order to check the validity of the proposed technique. The reason behind this choice was that such interval measures surely contain the unknown parameters with the uncertainty band of [− 3, 3%] subject to taking the pre-assumption of the deterministic uncertainty in the measurements within this range. It is essential to note that the proposed LCIs were followed as a result of considering the random behavior of the errors in the processing measurements rather than modeling such errors in a deterministic manner. Thus in the proposed method, uncertainty in the estimated parameters was quantified in terms of standard errors rather than describing the deterministic amount of plus minus 3% errors in the measurements. Although, the proposed approach and existence method were employed to estimate the interval bounds for the state parameters with different viewpoints about the error statistics but with the same intent. The results of the LCIs for the same unknown parameters of the 6-bus system have been evaluated for a sample of 28 observations since the large sample had a better performance in view of coverage probability for the linearized confidence intervals. The individual confidence intervals were evaluated at a nominal confidence level of 99% over 10,000 sets of simulations. This chosen level of significance with 17 degree of freedom raised to the corresponding quantiles of the t-distribution in the proposed method to approximately ±3. The plausible results of the LCIs besides those with the existing method of bounded intervals are reported in Table 10.

**Table 10:** The LCIs and bounded intervals for the unknown system state parameters of the 6-bus test network

Parameters to be estimated	True values	LCIs		Bounded intervals	
		Point estimates	Interval estimates	Point estimates	Interval estimates
*θ*_02_	−3.7	−3.67	(−3.99, −3.35)	−3.51	(−5.20, −2.52)
*θ*_03_	−4.3	−4.23	(−4.83, −3.71)	−3.91	(−5.88, −3.21)
*θ*_04_	−4.2	−4.19	(−4.52, −3.86)	−3.97	(−4.95, −3.09)
*θ*_05_	−5.5	−5.27	(−5.69, −4.85)	−4.84	(−5.10, −3.88)
*θ*_06_	−5.9	−5.94	(−6.50, −5.38)	−4.81	(−5.16, −1.47)
*V*_01_	241.5	241.53	(238.97, 244.08)	246.97	(239.59, 253.41)
*V*_02_	241.5	241.73	(239.17, 243.88)	245.59	(236.09, 250.88)
*V*_03_	246.1	246.13	(243.64, 248.62)	243.94	(235.70, 250.49)
*V*_04_	227.6	227.57	(224.92, 230.23)	231.33	(222.59, 237.38)
*V*_05_	226.7	226.67	(224.05, 229.30)	221.12	(216.08, 230.87)
*V*_06_	231.1	231.05	(228.40, 233.71)	231.15	(224.66, 239.45)

**Fig. 4:**
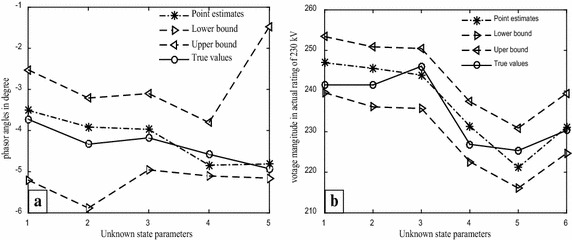
The uncertainty intervals for the system state parameters of the 6 bus system; **a** phasor angles, **b** voltage magnitudes

**Fig. 5:**
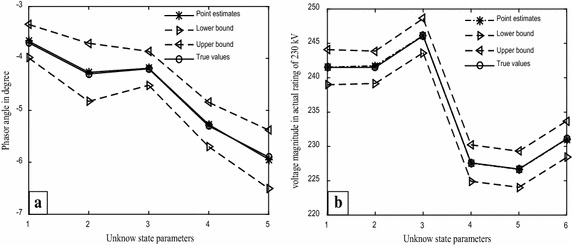
The LCIs for the system state parameters of the 6-bus system; **a** phasor angles, **b** voltage magnitudes

Clearly, the validation of the proposed method can be seen by the results presented in Table 10 as most of individual confidences intervals were within range of the bounded intervals, which surely contained the true system state parameters. Although, we have computed the linearized confidence interval for comparison purposes at a 99% confidence level due to having the identical margins of measurement errors, the same would confidently hold for the more narrow bounded intervals calculated in Tables 3 and 4 at 95%. Notice that the intervals, based on the proposed method representing the confidence bounds which may or may not constrain the unknown system parameters, and the probability of 95 or 99% confidence levels relate to the procedure reliability, not to an individual estimated interval. The comparative efficiency performance of the individual confidence intervals can be closely followed from the corresponding widths of the estimated intervals. To investigate this feature, interval estimates beside the point estimates from each method for the unknown system parameters of the 6-bus system are depicted in Figs. 4 and 5, respectively.

Figures 4 and 5 clearly exhibit that the proposed method considerably outperforms the uncertainty intervals since they provide the shortest widths and typically more uniform intervals in general. In addition, the point estimates of the proposed method seem to match with actual values due to the unbiasedness property of the least squares estimators.

## Conclusions

We have explored the problem in this paper by considering the random uncertainty in the measurements that ultimately originated uncertainty in the estimated parameters. In this way we have found the methodology to find the confidence intervals for the estimated parameters by deploying satisfactorily, the results of the nonlinear estimation theory along with the conventional state estimation algorithm. We have observed from our case studies, that the LCIs yield an adequate performance in the case of larger sample size. However, the lager sample size sample is not only the source of the adequate performance of the LCIs because of the nonlinearity of the estimated model. There may be several other sources of uncertainty that could contribute to the difficulty in calculating the optimum estimates and their asymptotic standard errors. Since, the output from the conventional state estimation algorithm for the point estimates is no more reliable in the presence of gross errors. In the same way, we have also been observed in our study, the poor performance of the LCIs in the presence of the gross error even for the larger sample size. This resulted in, an increase in the widths of the confidence intervals and hence a loss in the precision. Therefore, prior to the construction of the confidence intervals, consideration should be given to analyze the data in order to make sure that it is filtered by gross errors.

It is recommended to explore further the robust methods of interval estimation and their impact on the state estimation model that is presented in this study to further refine the computational technique.

## Abbreviations

WLS: weighted least squares
LCIs: linearized confidence intervals
ML: maximum likelihood
LCL: lower confidence limit
UCL: upper confidence limit
